# Performance comparison of VITEK MS PRIME with VITEK MS in clinical hospital laboratory setting

**DOI:** 10.1128/spectrum.01197-26

**Published:** 2026-08-19

**Authors:** Nina J. Gao, Paul M. Luethy, J. Kristie Johnson

**Affiliations:** 1 California Department of Public Health, Center for Laboratory Sciences 117025, Richmond, California, USA; 2 Department of Pathology, University of Maryland Baltimore School of Medicine 12264, Baltimore, Maryland, USA; University of Kentucky, Lexington, Kentucky, USA

**Keywords:** MALDI-TOF, microbial identification, diagnostics

## Abstract

MALDI-TOF mass spectrometry has been instrumental in rapid organism identification from clinical microbiology cultures. bioMérieux’s latest MALDI-TOF mass spectrometer for *in vitro* diagnostic laboratory use is the VITEK MS PRIME (MS PRIME). This instrument features a new internal multi-slide holder and streamlined sample acquisition. In this study, we performed a direct comparison between the MS PRIME and the VITEK MS for identification accuracy and time to result, to evaluate performance in a clinical laboratory setting. Two hundred fifty micro-organism isolates recovered from the University of Maryland Medical Center (UMMC) routine patient cultures were evaluated. Diagnostic accuracy for the MS PRIME was determined using the VITEK MS identification as the reference method. Organisms were considered correctly identified by the MS PRIME if an identification scored >99.0% confidence on at least two spots, and matched the VITEK MS identification. Slide loading and sample spot acquisition times were measured to compare functional time from slide preparation to receipt of final identification result for diagnostic reporting. The MS PRIME demonstrated high identification accuracy with 98.0% accuracy for all microbial isolates tested, except five failures from anaerobic bacteria (245/250). Average time to first sample acquisition from slide loading was 4 min 13 s for MS PRIME and 10 min 28 s for VITEK MS. Average spot acquisition time was significantly faster for MS PRIME (14 s, *n* = 100) compared to VITEK MS (25 s, *n* = 100). The MS PRIME demonstrates significantly faster time to results without significant loss in accuracy of microbial identification.

**IMPORTANCE:**

New instrumentation is often marketed as improved upgrades to existing *in vitro* diagnostic technologies. Our study evaluates the performance of the VITEK MS PRIME against its predecessor model in a high-throughput clinical diagnostic laboratory, putting the instrument to the test across a broad spectrum of microbial organisms. Our study not only looks at the technical and analytical performance of the instrument but also examines analyst hands-on time and workflow efficiency as points of consideration when evaluating a new instrument.

## INTRODUCTION

The adoption of matrix-assisted laser desorption ionization time-of-flight mass spectrometry (MALDI-TOF MS) in clinical diagnostic laboratories over a decade ago has dramatically reduced the time to definitive organism identification compared to biochemical reactions based on active growth. MALDI-TOF MS has significantly improved infectious disease diagnostics: colony identification has been reduced from 4 to 12 h using traditional biochemistry reactions and growth characteristics to minutes using a mass spectrometer with a reference mass spectra database (1–5).

MALDI-TOF MS for microbial identification varies based on organism and mass spectrometer model but generally comprises the following: isolated colony is grown or an organism is spotted on a target plate either directly or following protein extraction. Following the addition of a matrix solution, the prepared target plates are inserted into the mass spectrometer instrument where a laser creates ionized particles that are separated and detected on the basis of mass-to-charge ratios (sometimes denoted as *m/z*), creating unique spectra. The acquired mass spectra profiles of each unknown organism are compared to a well-characterized microbial database of known organisms to determine best matches for identification. Use of MALDI-TOF MS for organism identification has reduced microbiology laboratory operational costs after initial instrument adoption and with faster time to identification (1), leading to a significant reduction in time to appropriate diagnosis and antibiotic treatment (2, 3)—a highly valuable performance metric for patient care.

bioMérieux (Durham, NC, USA) has developed a new mass spectrometer model called the VITEK MS PRIME, an instrument designed to improve workflow and sample processing compared to the VITEK MS model, currently in wide use. The VITEK MS PRIME allows for continuous slide loading of up to 16 slides, unlike the predecessor, which requires manual operation and loading of up to only four slides at a time into the vacuum chamber; these improvements were adopted to speed up sample processing and reduce hands-on technical time required by a human operator (4). In addition, marketing discusses new components of the VITEK MS PRIME, including the laser and linear detector; these hardware changes may result in significant differences in performance between the two instruments. The charged ion spectra acquisition algorithm of the MS PRIME has also changed from the VITEK MS; the instrument no longer repeatedly fires at prepared spots to meet either a minimum threshold of quality spectra or until the spot sequence is completed; instead, a designated firing pattern is attempted on each spot for the VITEK MS PRIME. The change in acquisition programming can be a major factor toward time efficiency, especially for spots with little or no microbial material (i.e., negative control spots only with matrix) that may take minutes of spot acquisition laser firing in an attempt to recover spectra.

Although the MALDI-TOF MS time to organism identification is fast, especially compared to traditional culture-based and biochemical reaction-based methods, the hands-on processes in the laboratory are often slow. While some limitations cannot be improved with changes to spectrometer hardware, such as dependence on time and culture media for sufficient organism growth, the new VITEK MS PRIME is built for faster processing. The smaller single-slide capacity vacuum chamber is faster to depressurize and re-pressurize when accessing the chamber to load or unload slides. Though this may seem insignificant, this time delay was frequently cited as a complaint from human operators of the VITEK MS, especially in environments with high ambient relative humidity and subsequent longer vacuum purge time.

In this study, we sought to compare the performance of the VITEK MS PRIME to its predecessor, the VITEK MS, to determine the organism identification accuracy and operational efficiency in a clinical hospital laboratory setting.

## MATERIALS AND METHODS

### Culture and slide preparation of clinical isolates

Two hundred fifty isolates recovered from clinical specimens sent for routine diagnostic cultures at the University of Maryland Medical Center (UMMC) in Baltimore, Maryland, were used for this comparison study. All organisms were recovered on organism-appropriate solid agar-based media using standard-of-care methods verified for MALDI-TOF identification by the VITEK MS in the UMMC Clinical Microbiology Laboratory, and organism identifications are summarized in Table 1. Aerobic bacteria were recovered on tryptic soy agar with 5% sheep’s blood agar (SBA), chocolate agar, MacConkey agar, or phenylethyl alcohol blood agar (PEA) cultured at 35°C. Anaerobic bacteria were cultured using pre-reduced *Brucella* blood agar, PEA, laked kanamycin and vancomycin *Brucella* blood agar, or *Bacteroides* bile esculin agar cultured in GasPak EZ Pouch systems (BD) at 35°C. Yeasts were recovered on SBA or chocolate agar. Filamentous molds were recovered on Sabouraud dextrose agar or potato dextrose agar cultured at 25°C. Mycobacteria were recovered on 7H11 Middlebrook agar plates with or without selective antibiotics. Some organisms were cultured from frozen glycerol stocks originally recovered from patient cultures within the past 5 years for contemporary representation from diverse genera; these isolates were passaged twice in appropriate growth conditions prior to testing on both MALDI-TOF MS instruments.

**TABLE 1:** Summary of clinical isolates at UMMC used in this study

Reference identification	# ID	# ID (repeat)	# No ID	Total
Gram-positive aerobic bacteria	74	1	0	75
*Bacillus cereus* group	2	0	0	2
*Corynebacterium striatum^a^*	3	0	0	3
*Enterococcus faecalis*	9	0	0	9
*Enterococcus faecium*	5	0	0	5
*Glemia* species^*a*^^*a*^	1	0	0	1
*Granulicatella adiacens*	1	0	0	1
*Lactobacillus species*^*a*^	1	0	0	1
*Micrococcus luteus*	1	0	0	1
*Schaalia turicensis*^*a*^^*a*^	0	1	0	1
*Staphylococcus aureus*	16	0	0	16
*Staphylococcus epidermidis*	8	0	0	8
*Staphylococcus haemolyticus*	1	0	0	1
*Staphylococcus hominis*	1	0	0	1
*Staphylococcus saprophyticus*	1	0	0	1
*Staphylococcus simulans*	2	0	0	2
*Staphylococcus warneri*	1	0	0	1
*Streptococcus agalactiae*	2	0	0	2
*Streptococcus anginosus*	2	0	0	2
*Streptococcus dysgalactiae*^*b*^	1	0	0	1
*Streptococcus mitis/oralis*	1	0	0	1
*Streptococcus parasanguinis*	2	0	0	2
*Streptococcus pneumoniae*	2	0	0	2
*Streptococcus pyogenes*	5	0	0	5
*Streptococcus salivarius* group^*b*^	1	0	0	1
*Streptococcus sanguinis*	1	0	0	1
*Winkia neuii*	1	0	0	1
Gram-negative aerobic bacteria	71	0	0	71
*Citrobacter freundii* complex^*b*^	1	0	0	1
*Enterobacter cloacae* complex^*b*^	11	0	0	11
*Escherichia coli*	19	0	0	19
*Klebsiella oxytoca*	2	0	0	2
*Klebsiella pneumoniae*	15	0	0	15
*Neisseria gonorrhoeae*	2	0	0	2
*Proteus mirabilis*	7	0	0	7
*Proteus vulgaris*	1	0	0	1
*Pseudomonas aeruginosa*	5	0	0	5
*Pseudomonas stutzeri*	1	0	0	1
*Rothia mucilaginosa*	1	0	0	1
*Salmonella enterica*	1	0	0	1
*Serratia marcescens*	1	0	0	1
*Stenotrophomonas maltophilia*	3	0	0	3
*Vibrio vulnificus*	1	0	0	1
Anaerobic bacteria	15	5	5	25
*Bacteroides fragilis*	1	0	0	1
*Bacteroides ovatus/xylanisolvens*	1	0	0	1
*Bacteroides thetaiotaomicron*	1	0	0	1
*Bacteroides vulgatus*	0	1	0	1
*Bilophila wadsworthia*	1	0	0	1
*Campylobacter ureolyticus*	1	1	0	2
*Clostridium clostridioforme*	0	0	1	1
*Clostridium perfringens*	1	0	0	1
*Clostridium tertium*	1	0	0	1
*Cutibacterium acnes*	1	0	0	1
*Eggerthella lenta^a^*	0	0	1	1
*Finegoldia magna*	0	1	0	1
*Fusobacterium nucleatum*	2	0	0	2
*Parabacteroides* species^*a*^	1	0	1	2
*Parvimonas micra*	1	2	0	3
*Peptoniphilus assacharolyticus*	1	0	0	1
*Peptoniphilus indolicus*	0	0	1	1
*Prevotella bivia*	1	0	0	1
*Prevotella denticola*	1	0	0	1
*Prevotella melaninogenica*	1	0	0	1
*Prevotella oris*	1	0	0	1
*Veillonella* species^*a*^^,^^*b*^	1	0	1	2
Yeast	23	2	0	25
*Candida albicans*	8	2	0	10
*Candida dubliniensis*	1	0	0	1
*Candida glabrata*	4	0	0	4
*Candida krusei*	2	0	0	2
*Candida parapsilopsis*	3	0	0	3
*Candida tropicalis*	4	0	0	4
*Cryptococcus neoformans*	1	0	0	1
Filamentous fungi (mold)	26	2	0	28
*Alternaria alternata*	1	0	0	1
*Aspergillus fumigatus*	4	0	0	4
*Aspergillus nidulans*	1	0	0	1
*Aspergillus niger* complex	1	0	0	1
*Aspergillus sydowii*	1	0	0	1
*Aspergillus terreus* complex	1	0	0	1
*Curvularia spicifera*	1	0	0	1
*Exophiala dermatitidis*	1	0	0	1
*Fusarium oxysporon* complex	1	0	0	1
*Fusarium solani* complex	2	0	0	2
*Microsporum canis*	1	0	0	1
*Paecilomyces variotii* complex	1	0	0	1
*Penicillium camemberti*	1	0	0	1
*Penicillium chrysogenum*	0	1	0	1
*Pseudallescheria boydii*	1	0	0	1
*Purpureocillium lilacinum*	1	0	0	1
*Rasamsonia argillacea*	1	0	0	1
*Rhizopus arrhizus* complex	1	0	0	1
*Rhizopus microsporus* complex	1	0	0	1
*Sporothrix schenckii* complex	2	0	0	2
*Trichophyton interdigitale*	0	1	0	1
*Trichophyton rubrum*	2	0	0	2
Acid-fast bacilli (mycobacteria)	25	1	0	26
*Mycobacterium abscessus*	3	1	0	4
*Mycobacterium avium* complex	5	0	0	5
*Mycobacterium chelonae*	1	0	0	1
*Mycobacterium fortuitum* group	1	0	0	1
*Mycobacterium gordonae*	3	0	0	3
*Mycobacterium haemophilum*	1	0	0	1
*Mycobacterium marinum*	2	0	0	2
*Mycobacterium simiae*	1	0	0	1
*Mycobacterium tuberculosis* complex	5	0	0	5
*Mycobacterium xenopi*	3	0	0	3

^*a*^ Denotes organism ID that is not FDA-validated in VITEK v3.2.0 Knowledge Base, but internally validated at UMMC to species or genus level.

^*b*^ Denotes organism ID found acceptable to a species complex level due to split confidence for similar valid species or subspecies identifications.

All bacterial isolates were prepared from pure colony growth onto VITEK MS-DS slides (bioMérieux) using the VITEK MS PICKME pen and disposable nibs (bioMérieux) with alpha-cyano-4-hydroxycinnamic acid (CHCA) matrix overlay. Yeast colonies were also prepared using the PICKME except colony growth was first prepared with formic acid overlay followed by CHCA matrix. Filamentous fungi and mycobacteria were extracted and prepared according to the manufacturer’s instructions using the VITEK MS Mold Kit (bioMérieux) and VITEK MS Mycobacterium/Nocardia Kit (bioMérieux), respectively. For a fair comparison, all slide spots in this study were prepared in parallel for both the VITEK MS and VITEK MS PRIME by the same operator on the same day from the same agar growth. Use of clinical isolates was deemed as exempt by the University of Maryland Baltimore IRB under protocol number HP-00104589.

### Organism identification

Organism identification by the VITEK MS-acquired spectra using the VITEK v3.2.0 IVD Knowledge Base (bioMérieux) served as the reference standard for this study. Identifications on the VITEK MS PRIME with “Good identification” (noted as confidence >60–99.9%) as determined by VITEK MS software from two spots were considered acceptable identification. All reference identifications were generated on the first attempt on the VITEK MS, with no isolate requiring repeat testing to determine a “Good identification” by the VITEK software. All isolates were initially tested with two target spots; failure to achieve “Good identification” across two spots warranted repeat testing with an additional two spots. Repeat testing with “Good identification” for the same organism ID from at least two of the four spots was considered acceptable identification upon repeat. Any organisms that failed to yield an identification on the VITEK MS PRIME despite repeat testing resulted in failure to identify. Identifications called at different levels (i.e., genus vs species-level identification) or species-level identifications that are routinely reported as a species complex or at the genus level by our laboratory were not considered discrepant. Organisms not included in the FDA-validated IVD Knowledge Base were internally validated at UMMC. The internal validation process includes confirmation of organism identification for three distinct clinical isolates through an alternative method (e.g., sequencing). Refer to Table S1 for details on species complexes and identifications internally validated at UMMC Clinical Microbiology Laboratory.

### Timing studies for workflow efficiency

Timing studies were performed using the stopwatch function on digital timers. Slide loading time was defined as the time taken by a technologist to load a MS-DS slide into the mass spectrometers. For VITEK MS, slide loading time was calculated from opening the vacuum chamber for access to the start of the first spot acquisition. For VITEK MS PRIME, slide-loading time was from the insertion of the prepared slide into the puck to the start of the first spot acquisition. Spot acquisition time was determined by timing the duration of spectra acquisition from individual spots prepared in parallel by the same technologist for fair comparison. VITEK MS PRIME allows for defined settings; our instrument was set to allow up to three acquisition attempts per sample spot to acquire sufficient spectra. Therefore, spot acquisition time on the VITEK MS PRIME was defined including all attempts (up to three). Statistical analyses and figure generation were performed in RStudio version 2021.9.0.351 using R version 4.1.1 (5, 6) and the tidyverse R package version 1.3.1 (7).

## RESULTS

To evaluate the performance of the VITEK MS PRIME compared to the existing VITEK MS, we sought to use microbial isolates recovered during routine culture in the academic hospital laboratory setting that ensured representation from gram-positive aerobic bacteria, gram-negative aerobic bacteria, anaerobic bacteria, yeasts, filamentous fungi (molds), and acid-fast bacilli (AFB, mycobacteria) recovered on appropriate solid media. All isolates used for the comparison study and results of identification are summarized in Table 1. All organisms were tested for at least two spots using the recommended identification method; identification that matches the reference identification from the first attempt is denoted as “ID” on Table 1. Any isolates that initially failed to ID were repeated; subsequent successful identification was noted separately and is noted on Table 1 as “ID (repeat).” Any isolates that do not meet our acceptance criteria for identification after a total of four spots were marked as failure to identify by the VITEK MS PRIME. Across all representative organisms tested, the VITEK MS PRIME succeeded in identifying 93.6% (234/250) of clinical isolates on the first attempt, increasing to 98.0% (245/250) including success with repeat testing (total of four spots). All groups of organisms tested exceeded 92.0% successful identification on the first attempt except for anaerobic bacteria; only 60.0% (15/25) of anaerobic bacteria isolates tested were identified with the first two spots, increasing to only 80.0% (20/25) with repeat testing. No isolates were misidentified by the VITEK MS PRIME in this study compared to the reference identification on the VITEK MS.

**Fig 1:**
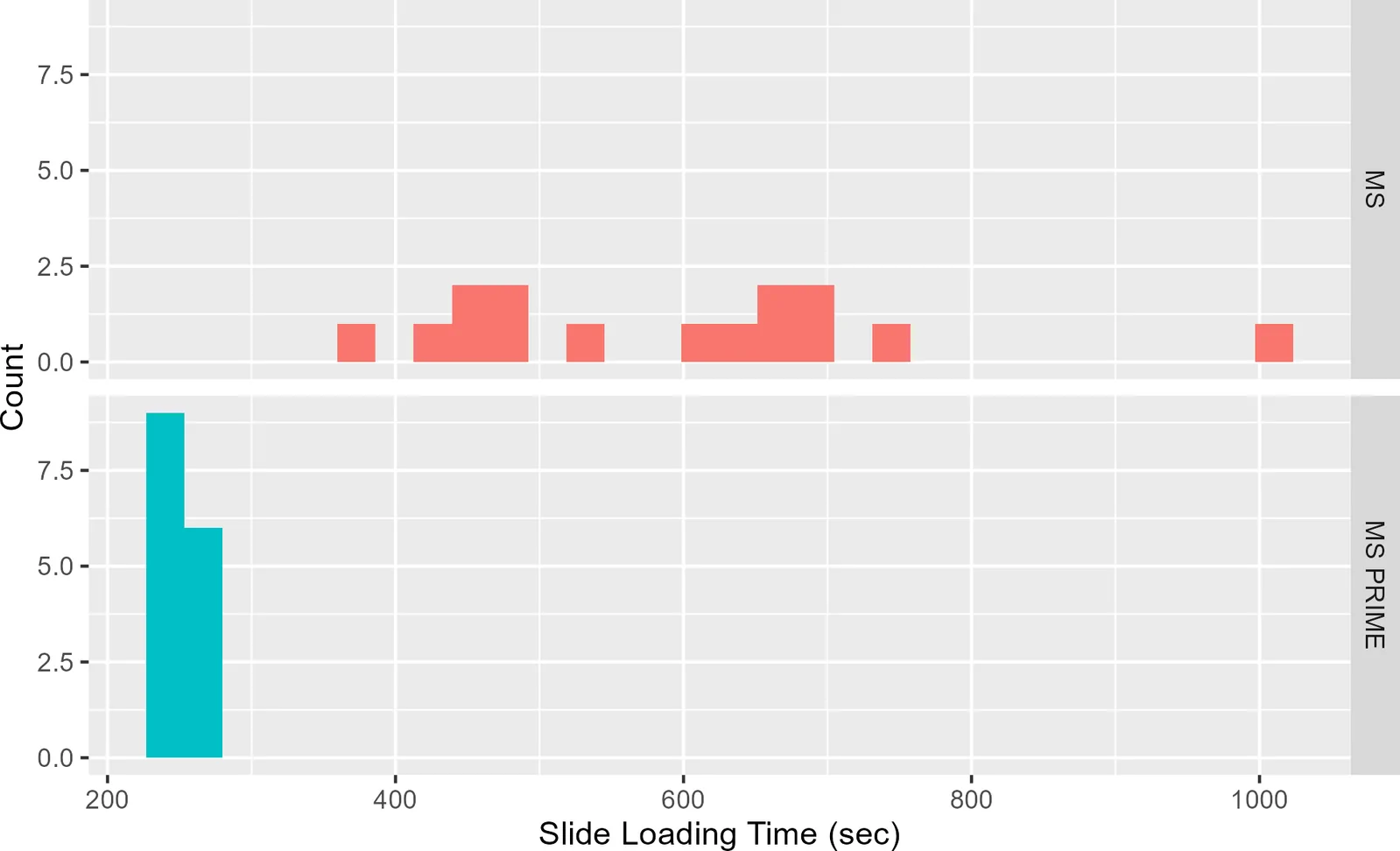
Histograms of time to load MS-DS slides onto VITEK MS (MS) compared to VITEK MS PRIME (MS PRIME). *N* = 15 slides loaded onto each instrument. Vertical dashed line visualizes arithmetic mean (592 s for MS and 252 s for MS PRIME). *P* = 3.356 × 10^−6^ by Wilcoxon rank-sum test with continuity correction.

To evaluate workflow efficiency in a clinical laboratory, we timed the performance of the VITEK MS and VITEK MS PRIME for the time it took trained technologists to both load an MS-DS slide into the mass spectrometers and time for spectra acquisition for individual spots, as these are key factors to determine time to identification result. The slide loading time for the VITEK MS PRIME was significantly and consistently shorter than the VITEK MS, averaging only 252 s compared to 592 s, respectively (Fig. 1, *P* = 3.356 × 10^−6^).

**Fig 2:**
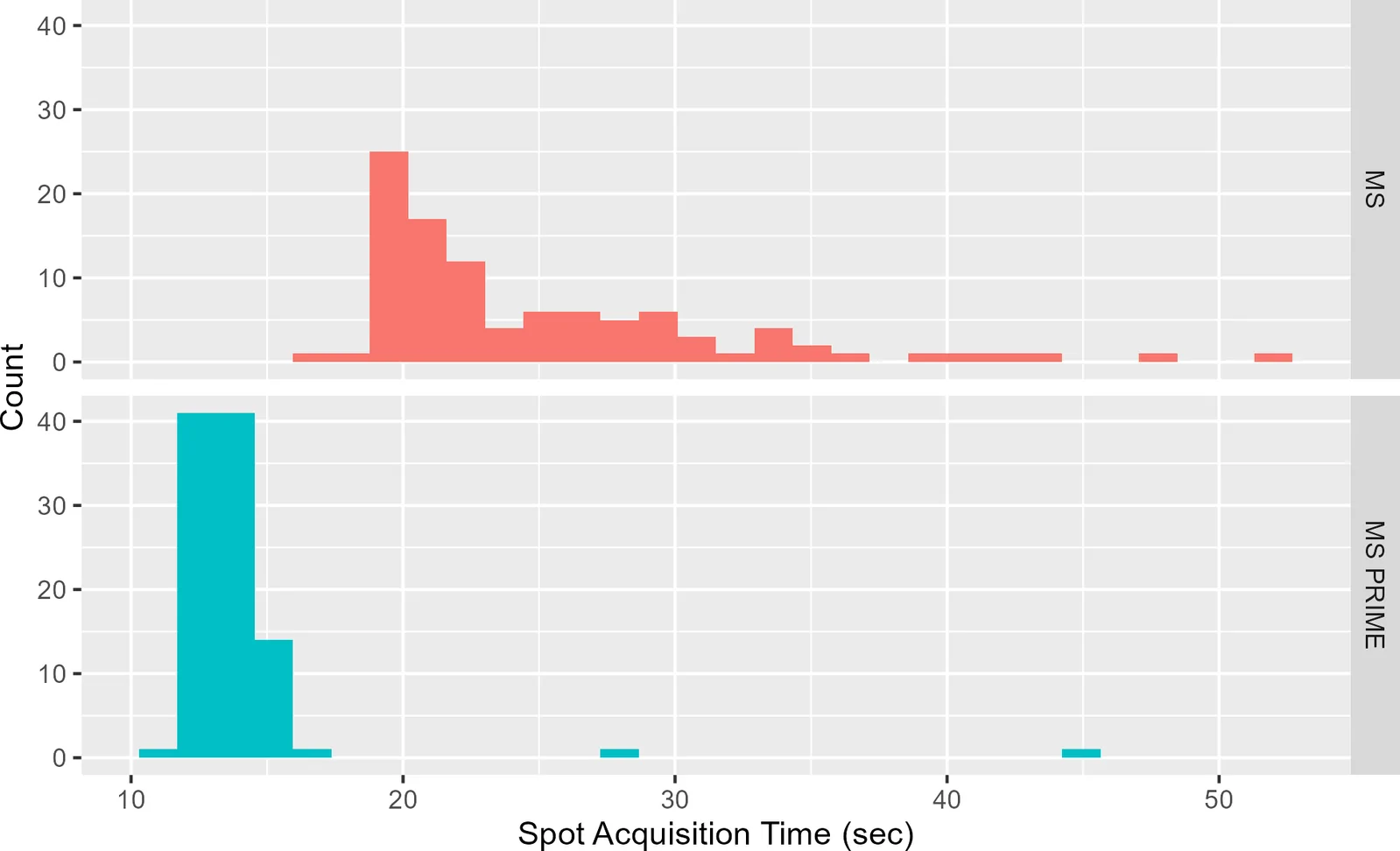
Histograms of spot acquisition time from individual MS-DS sample spots for VITEK MS (MS) compared to VITEK MS PRIME (MS PRIME). Vertical dashed line visualizes mean for each instrument (25.0 s for VITEK MS and 14.1 s for VITEK MS PRIME). *N* = 100 spots for each instrument. *P* < 2.2 × 10^−16^ by Wilcoxon rank-sum test with continuity correction.

The acquisition of charged ion spectra from the VITEK MS PRIME has changed from the VITEK MS. We timed the duration of acquisition from individual prepared sample spots to test if there was a significant difference (Fig. 2). Programming for spot acquisition between the two instruments is different: for the VITEK MS, laser firing and sample acquisition continue along a serpentine pattern across the sample spots until sufficient acceptable profiles are acquired for identification, a process taking up to 52 s for a single spot. In contrast, the VITEK MS PRIME was set to allow up to three acquisition attempts per sample spot, resulting in a tri-modal distribution of acquisition times. Each acquisition attempt averaged approximately 14 s, albeit most sample spectra were acquired upon the first attempt (Fig. 2, lower panel). On average, total spot acquisition time was significantly faster (*P* < 2.2 × 10^−16^) on the VITEK MS PRIME (average: 14.1 s) compared to the VITEK MS (25.0 s).

## DISCUSSION

MALDI-TOF MS identification for clinical diagnostic use has delivered fast time to identification without loss of accuracy so long as the organism spectra were part of the validated databases. The technology boasts high accuracy across an ever-broadening spectrum of organisms with regular updates and improvements to identification databases (8–12). The VITEK MS PRIME brings promise of yet faster processing time with the adoption of a smaller vacuum chamber for faster depressurization. The range of slide loading time for the VITEK MS spans many minutes, from as short as 368 s (6 min 8 s) to as long as 1010 seconds (16 min 50 s), with the longest time due to the prolonged duration that the vacuum slide chamber was left open as the operator removed the completed slides and loaded the newly prepared slides into the slide holder. While we initially considered dismissing this observation as an outlier, as this occurred just before routine preventative maintenance, we ultimately decided to keep this observation point in the study as it reflects the reality of long processing times for the VITEK MS and the impact it has on the time to identification due to the reliance of human operators. Conversely, the VITEK MS PRIME independently controls the opening and closing of the vacuum chamber instead of relying on human operators, allowing for faster processing time. Although the VITEK MS PRIME only processes a single prepared slide at a time, a smaller capacity compared to the four-slide capacity of the VITEK MS slide holder, the operator-free workflow uses a robotic arm to transfer slides in and out of the vacuum chamber and onto a slide holding rack with a maximum capacity of 16 slides. This setup allows for continuous, operator-free slide acquisition of all loaded slides without human intervention, a significant improvement to workflow and efficiency without requiring manual manipulation for each batch of slides awaiting analysis. Automated continuous operation on the VITEK MS PRIME allows for shorter hands-on and wait time for technologists performing the same task of loading slides for MALDI-TOF mass spectrometry, improving efficiency in the laboratory by reducing the duration of time spent idling next to the instrument, waiting for vacuum chamber pressurization and/or depressurization while loading slides for analysis. Overall time to organism identification was significantly faster using the VITEK MS PRIME than the VITEK MS, with some slide acquisition groups completing and reporting organism identifications from the VITEK MS PRIME before the first spot acquisition had begun on the VITEK MS instrument loaded at the same time. Though the time savings for both spot acquisitions and slide loading may only be a few seconds and seem insignificant, large laboratories performing hundreds of organism identifications each day and around the clock can gain significant improvement in continuous operation. The hands-free time afforded by the VITEK MS PRIME could result in improved work efficiency and morale for the technical staff, as staff are no longer required to wait at the mass spectrometer; instead, staff may use the time to perform other tasks.

The VITEK MS PRIME performed well in the identification of most clinical isolates tested, with over 90% identification to the organism level across all organism types tested (Table 1). Note that one limitation of our study is the omission of *Nocardia* species and other actinomycetes, as this was not yet a validated organism group for our MALDI-TOF MS workflow, and therefore we were unable to evaluate identification performance. We expected some potential challenges with MALDI-TOF MS identification of molds and acid-fast bacilli, as these have historically been difficult organisms to identify (11, 12); however, our study shows that the performance of the VITEK MS PRIME is comparable to VITEK MS when the appropriate protein extraction kits are used from colony growth on solid agar. MALDI-TOF MS identification of anaerobic bacteria, however, did not fare as well. Identification of anaerobic bacteria by MALDI-TOF MS has proved challenging, with reported identification rates as low as 75% in early publications (13). However, this low identification success rate was largely attributed to limitations of early spectra databases, with increased identification of anaerobic species with each database expansion and update (10). The database is unlikely to be the cause of identification misses for the VITEK MS PRIME, as both instruments in this study use the same software and analysis pipeline with VITEK MS v3.2.0 Knowledge Base, although they differ in acquisition of charged ion spectra from parallel slide preparations. The MS PRIME does not allow for re-firing of previously run slides, another limitation to the study where the same slide preparations cannot be tested across both instruments to test spectra acquisition. It is also possible that the programming for spectra acquisition for the VITEK MS PRIME is optimized for shorter acquisition time and performs well for most organisms but falls short of sufficient spectra acquisition for anaerobic bacteria, which can be trickier to distinguish. Spot acquisition of anaerobes on the VITEK MS may have resulted in longer duration to acquire more mass spectra in exchange for subsequent successful identification due to the different acquisition pattern. It is possible that protein extraction may improve identification of anaerobic bacteria on the VITEK MS PRIME, as there were no issues in the identification of the extracted filamentous fungi nor AFB using the appropriate kits, despite known challenges with MALDI-TOF MS identification of those organism groups. Further investigation is warranted for the identification accuracy of anaerobic organisms on the VITEK MS PRIME, especially as lower accuracy for anaerobic bacterial identification was not observed in another study (4). A separate study dedicated to the identification of 60 anaerobic bacterial isolates (14) found similar identification discrepancies between the VITEK MS PRIME and Biotyper Sirius. However, poor resolution of select taxa was observed in the VITEK MS PRIME compared to the Biotyper Sirius, suggesting that identification differences may be due to spectra databases rather than hardware or loss of quality spectra acquisition due to the faster and abbreviated acquisition program. The improvement in speed may have resulted in a loss of accuracy for challenging-to-identify organisms, such as certain genera of anaerobic bacteria.

Overall, the VITEK MS PRIME has significantly improved the speed of microbial identification in the clinical laboratory by operating continuously and independently of human operators when loading and unloading slides into the vacuum chamber for analysis. This improved efficiency not only shortens the time to a definitive organism identification, which is critical for proper diagnosis and treatment of infectious disease, but also improves the laboratory workflow, giving time back to the technologists to perform other tasks away from the mass spectrometer.

## Data Availability

The full list of de-identified isolates, identifications from both mass spectrometers, repeat testing, and R scripts written to generate the figures are available from authors upon request.

## SUPPLEMENTAL MATERIAL

Supplemental materials spectrum.01197-26-s0001.docx Supplemental methods and Table S1.

ASM does not own the copyrights to Supplemental Material that may be linked to, or accessed through, an article. The authors have granted ASM a non-exclusive, world-wide license to publish the Supplemental Material files. Please contact the corresponding author directly for reuse.
