# Supplementary material for: Performance comparison of VITEK MS PRIME with VITEK MS in clinical hospital laboratory setting
Source: Microbiol Spectr. 2026 Aug 19;14(10):e01197-26. doi: 10.1128/spectrum.01197-26 (PMC13689162; doi:10.1128/spectrum.01197-26)
Supplement: Supplementary file 1 [file spectrum.01197-26-s0001.docx]

**Supplemental Material**

**Supplemental Method**: Internally validated organism identifications and acceptable split identifications

Our study includes several organism identifications that are part of VITEK® v3.2 Knowledge Base but are not claimed by FDA validation but were internally validated at UMMC using at least 3 unique isolates with definitive identification using alternative methods (i.e. 16s sequencing, MALDI-TOF identification at a reference laboratory, biochemical panels). These internally validated identifications were determined to be valid to either the genus or species identification level after director review and are summarized in Supplemental Table 1.

Any MALDI-TOF spectra spots that yielded a split identification (frequently evenly split as 50/50 confidence between two identifications or 33.3/33.3/33.3 confidence split between 3 identifications) were reviewed for acceptability. Species complexes and genera known to have similar mass spectra in the Vitek Knowledge Database are acceptable identifications and are detailed in Supplemental Table 1 below.

**Supplemental Table 1. Internally validated organism ID’s and acceptable species complexes / groups used in this study**

| Mass spectrometer ID | UMMC Validated ID |
| --- | --- |
| *Arcanobacterium haemolyticum* {N} | *Arcanobacterium haemolyticum* |
| *Campylobacter ureolyticus* {N} | *Campylobacter ureolyticus* |
| *Citrobacter braakii,  Citrobacter freundii* | *Citrobacter freundii* complex |
| *Corynebacterium striatum* {N} | *Corynebacterium striatum* |
| *Eggerthella lenta* {N} | *Eggerthella lenta* |
| *Enterobacter asburiae,  Enterobacter cloacae,  Enterobacter hormachei, Enterobacter hormachei* subsp*. hormachei, Enterobacter ludwigii* | *Enterobacter cloacae* complex |
| *Gleimia europaea* {N} | *Gleimia* species |
| *Lactobacillus casei/rhamnosus/paracasei* {N} | *Lactobacillus* species |
| *Mycobacterium avium,  Mycobacterium intracellulare* | *Mycobacterium avium* complex |
| *Parabacteroides distasonis* {N} | *Parabacteroides* species |
| *Schaalia turicensis* {N} | *Schaalia (Actinomyces) turicensis* |
| *Streptococcus dysgalactiae* ssp. *dysgalactiae, Streptococcus dysgalactiae* ssp *equisimili* | *Streptococcus dysgalactiae* |
| *Streptococcus salivarius ssp. salivarius, Streptococcus salivarius ssp. thermophilus, Streptococcus vestivularis* | *Streptococcus salivarius* group |
| *Veillonella atypica* {N}*,  Veillonella parvula* {N} | *Veillonella* species |

{N}: Denotes mass spectrometer ID’s that are not FDA-validated in the VITEK® MS v3.2.0 Knowledge Base
